# Real-world testing of the durability claims of a commercially available spray-on surface biocide

**DOI:** 10.1128/aem.00803-25

**Published:** 2025-09-15

**Authors:** Shilpa Saseendran Nair, Alana Cavadino, Simon Swift, Siouxsie Wiles

**Affiliations:** 1 Department of Molecular Medicine and Pathology, Waipapa Taumata Rau University of Auckland 1415, Auckland, New Zealand; 2 Department of Epidemiology and Biostatistics, Waipapa Taumata Rau University of Auckland 1415, Auckland, New Zealand; Centers for Disease Control and Prevention, Atlanta, Georgia, USA

**Keywords:** transmission, fomites, quat, antibacterial surfaces, continued action, residual action, biocide, surface-anchoring

## Abstract

Self-disinfecting biocidal surfaces have been proposed as a solution to prevent infections caused by the transmission of microorganisms from frequently touched surfaces in hospitals and other settings. For example, surface anchoring quaternary ammonium salt (SAQAS)-based biocides are purported to maintain their antimicrobial potency for up to 30 days and are available as spray-on formulations. We tested the 30-day potency claim of a commercially available spray-on SAQAS-based biocide (“SAQAS-A”) under “real-world” conditions in a microbiology laboratory where regular cleaning is routine. To do this, we determined the background microbial burden on high-traffic floor areas, routinely used bench areas, frequently touched handles, and glass surfaces for 30 days before and after applying SAQAS-A. Experiments were repeated three times, and data were analyzed using two generalized linear mixed models (GLMM). We observed that for >80% of bench samples, the number of viable bacteria recovered was below the highest acceptable level of 2.5 colony-forming units (CFU)/cm^2^, with minimal reduction in CFU recovery observed after SAQAS-A treatment. In contrast, the number of floor and glass samples in which the microbial burden exceeded 2.5 CFU/cm^2^ was greater after biocide application (12.7% and 73%, respectively) than before (4.8% and 37%, respectively). Analysis of all data using two statistical models confirmed that the application of SAQAS-A had no antimicrobial effect either after five or 30 days of application. In conclusion, our results indicate that SAQAS-A was ineffective in preventing surface contamination over 30 days in a real-world scenario where routine cleaning occurs.

**IMPORTANCE:**

We tested the claim that a commercially available spray-on biocide could protect surfaces from microbial contamination for 30 days. In the presence of routine disinfection, we found that spray-on applications of the surface anchoring quaternary ammonium salt-based biocide did not protect frequently touched surfaces in a working microbiology research lab for up to 30 days. Our data show that users of this type of product should be aware of the limitations of manufacturers' claims of “continued protection”, and manufacturers should consider formulations that more reliably attach to surfaces. Similarly, regulatory and consumer protection agencies should provide clear guidance for companies wishing to make durability and longevity claims for biocidal surface applications.

## INTRODUCTION

It is now well established that surfaces play a role in the transmission of pathogens to patients within hospitals (1, 2). Frequently touched surfaces, such as medical equipment and items in the hospital environment (for example, bed rails), act as a reservoir for bacteria and viruses (3, 4) and can lead to hospital-acquired infections with severe outcomes (5–7). So, while environmental transmission via fomites is a problem, it also presents an opportunity for control if reservoirs can be eradicated or routes of transmission broken. Environmental cleaning and surface disinfection are important hygiene measures that contribute to minimizing the transmission of hospital-acquired pathogens (8, 9). However, there are several drawbacks associated with the currently available strategies. These include variations in bactericidal efficacy, microbial resistance, cost, non-eco-friendly chemical constituents, and the likelihood of recontamination. Standard operating procedures for cleaning and decontamination also differ within and between hospitals (9). These drawbacks have driven the development of alternative technologies, such as “self-disinfecting” biocidal surfaces (10, 11).

Ideally, biocidal surfaces should exhibit durable, broad-spectrum antimicrobial activity and be made from biocides that are biodegradable and non-toxic to humans and the environment, thereby avoiding long-term environmental persistence. One strategy to create such surfaces is to anchor biocidal agents onto the surface, enabling microbes to be killed on contact. An advantage of this strategy is that it is more eco-friendly as it requires smaller amounts of the active compound, either incorporated into the material or applied onto the material surface (12–14), and the antimicrobial agent is retained without leaching into the environment. However, limitations include the durability of coatings on different surfaces, the emergence of resistant strains after prolonged periods of continuous exposure, and microorganisms associated with particles not coming into lethal intimate contact with the biocide (15, 16).

The antimicrobial activity of 3-(trimethoxysilyl)-propyldimethyloctadecyl ammonium chloride, an organosilicon quaternary ammonium compound, has been commercialized for use as a surface sanitizer. The commercial products are manufactured in spray formulations that are sold ready to use at room temperature (17). Marketing materials claim that these “hospital-grade” products are durable, exhibit broad-spectrum antimicrobial activity, and are non-toxic to human cells, making them eco-friendly. According to the manufacturers, when applied, these biocides become covalently attached to the surface and polymerize to form a stable and long-lasting coating (17). After application, the biocide dries onto the surface and is proposed to form a thin coating of molecular spike-like structures at the surface, which electrostatically attract microorganisms, killing them by rupturing the cell envelope. Hence, it is argued that the possibility of resistant strains evolving is reduced because this compound kills mechanically. In some cases, it is claimed that the biocide is stable and can ensure protection for around 1 month, preventing recontamination in that period, even in the presence of regular cleaning (18). Several studies have evaluated the efficacy of quaternary ammonium compound sprays, but typically only for a short period after application (for example, 2–24 h) (19–21).

The international standards by which the potency of antimicrobial surfaces is often measured (for example, the Japanese Industrial Standard Z 2801:2012 and International Organisation of Standardisation [ISO] 22196:2011 [22]) have not covered the aspect of long-term durability, and therefore, regulatory and consumer protection agencies are limited in their ability to provide clear guidance for companies seeking to make claims of durability and longevity for biocidal surface applications. New standards that incorporate tests of the durability of antimicrobial treatments, such as PAS 2424:2014 (23), are a step in this direction. Indeed, in response to the COVID-19 pandemic, the United States Environmental Protection Agency (EPA) updated its guidance for companies seeking to claim residual activity for their products, as reviewed by Donskey (24). Importantly, this guidance includes a method to simulate cycles of in-service disinfection and cleaning (25). Companies may also be able to obtain EPA approval for a 24 h residual antimicrobial activity claim based on standard testing. However, these tests only simulate the ability to withstand routine touching and handling, and the products must be reapplied after cleaning. On its website, the EPA lists residual antimicrobial products approved for use against severe acute respiratory syndrome coronavirus 2 (26). At the time of writing, the list was last updated on 22 April 2025 and contains only two products, both of which are copper-containing paints.

In this study, we tested the 30-day activity/durability claims of a commercially available surface anchoring quaternary ammonium salt (SAQAS)-type spray-on biocide that we have previously shown to be active against model Gram-positive and Gram-negative species and antibiotic-resistant isolates when applied to glass and low-density polyethylene surfaces (27). We tested the 30-day activity/durability claims in a Physical Containment (PC) 2 microbiology laboratory, equivalent to Biosafety Level 2, as a surrogate for controlled, high-traffic environments such as hospitals where regular cleaning occurs. We regularly swabbed designated laboratory areas before and after applying a commercial SAQAS product (“SAQAS-A”) according to the on-label instructions. The generally acceptable burden of environmental bacteria on hospital surfaces is 2.5 colony-forming units (CFU)/cm^2^ (28–30), so we used this value to measure the success of the surface treatment. We demonstrate that SAQAS-A was unable to protect surfaces for 30 days, as claimed by the manufacturer (18), in a real-world scenario that included routine chemical disinfection of surfaces.

## RESULTS AND DISCUSSION

**Fig 1:**
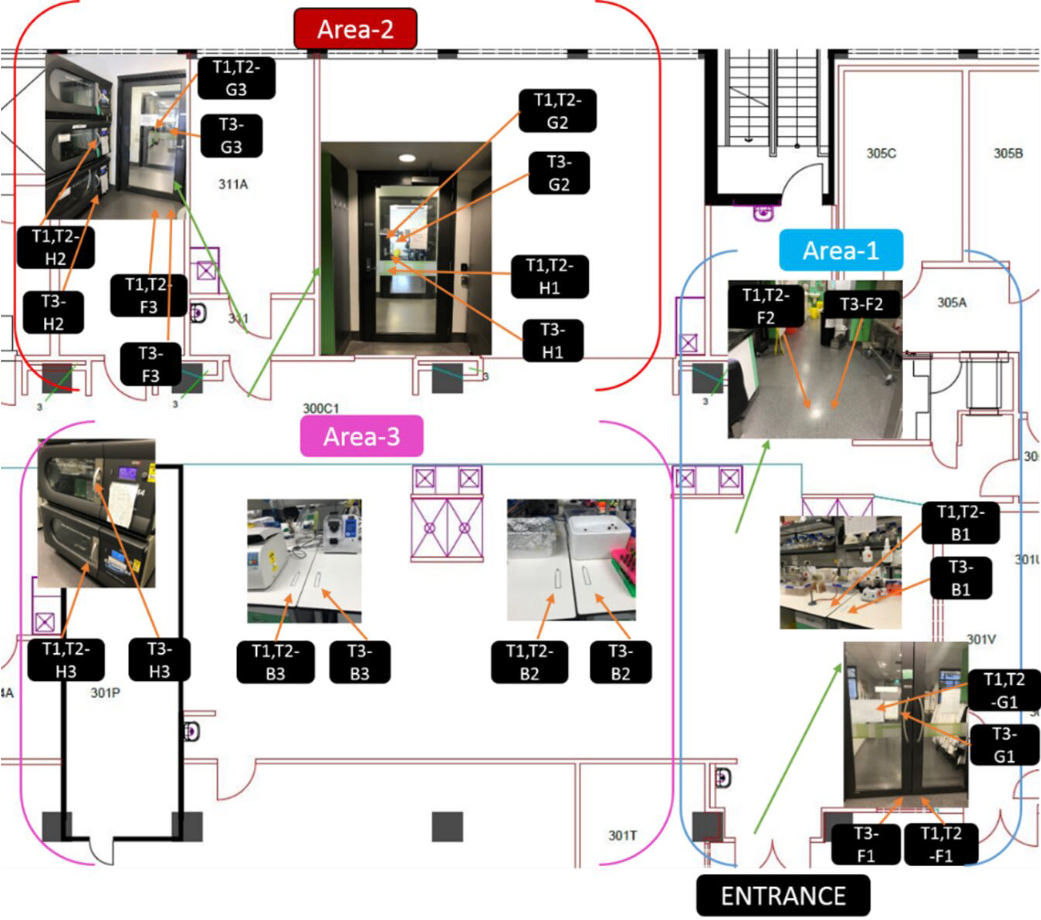
Laboratory areas chosen for real-world testing. The laboratory was divided into three areas as indicated on the plan. Key: B, bench; F, floor; G, glass; H, handle; T1, trial 1; T2, trial 2; T3, trial 3.

We have previously shown that SAQAS-A is active against model Gram-positive and Gram-negative species when applied to glass and low-density polyethylene surfaces (27). In this study, we evaluated the manufacturer’s durability claims for SAQAS-A by swabbing various laboratory surfaces (each with a sampling area of 20 cm^2^) for 30 days before and after SAQAS-A application and recovering any bacteria present on selective agar. We selected three high-traffic floor areas, three routinely used bench areas, three frequently touched handles, and three glass surfaces as sampling sites (Fig. 1) and conducted three independent trials. Of the many species recovered (see Table S2 at https://doi.org/10.17608/k6.auckland.29983177), only *Pseudomonas putida* has previously been used by researchers within the laboratory. We did not test whether the species recovered were intrinsically more resistant to SAQAS-A than the model organisms previously examined.

### SAQAS-A did not offer an advantage as a biocidal treatment for routinely decontaminated or infrequently contaminated surfaces

**Fig 2:**
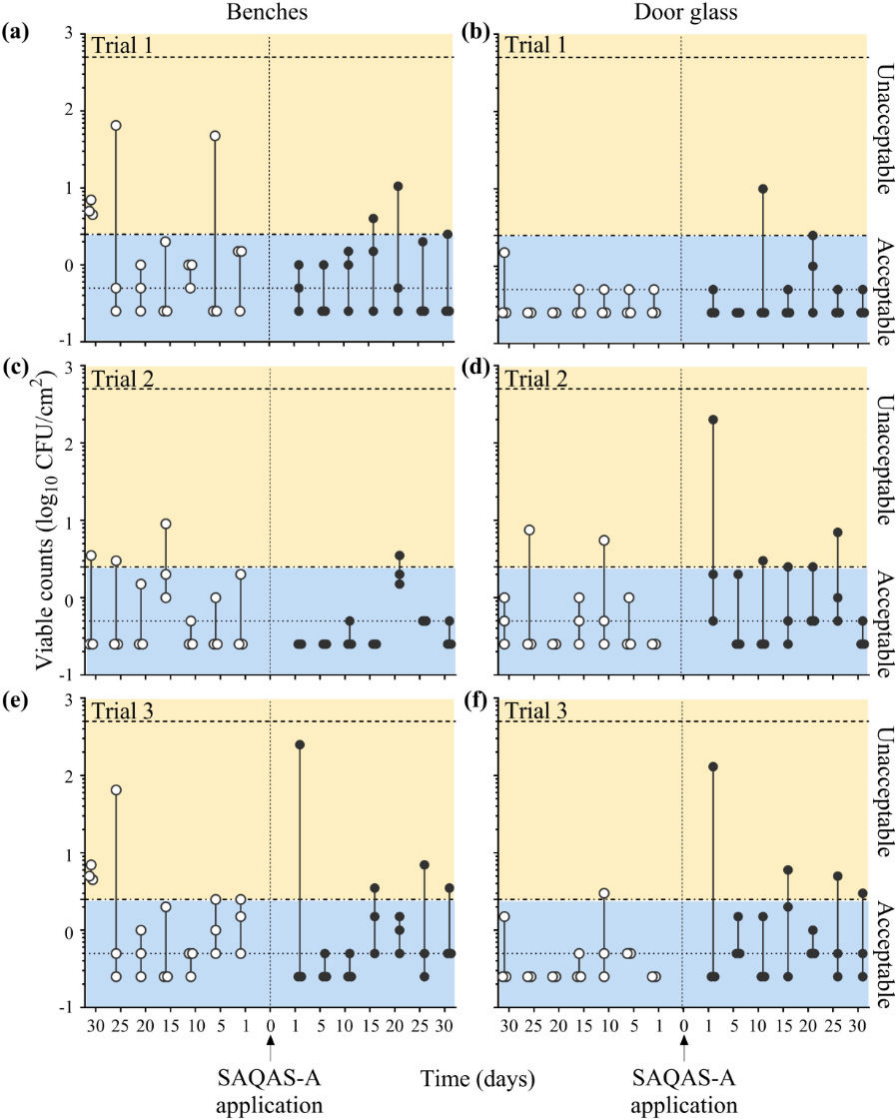
Bacterial counts recovered from benches and door glass before and after the application of SAQAS-A. Data presented as individual viable counts (log_10_ CFU/cm^2^) recovered at regular intervals for 30 days before (open circles) and after (closed circles) the application of SAQAS-A to benches (a/c/e) and glass areas (b/d/f). Experiments were performed over three trials (Trial 1 [**a/b**], Trial 2 [**c/d**], and Trial 3 [**e/f**]). Dotted lines indicate the upper and lower limits of detection. Blue boxes (labeled “Acceptable”) denote viable counts below the acceptable level of 2.5 CFU/cm^2^; yellow boxes (labeled “Unacceptable”) denote viable counts above this level.

Our data show that the number of viable bacteria recovered from most laboratory benchtops and door glass samples was below the highest acceptable level of 2.5 CFU/cm^2^, with minimal reduction in CFU recovery observed after biocide treatment (Fig. 2). For laboratory benches, 12/63 (19%) of samples (5/21 [Trial 1], 3/21 [Trial 2], and 4/21 [Trial 3]) exceeded the acceptable level before treatment, while 7/63 (11%) of samples (2/21 [Trial 1], 1/21 [Trial 2], and 4/21 [Trial 3]) exceeded the acceptable level after treatment (Fig. 2a, c, and e). Similarly, 3/63 (4.8%) of door glass samples (0/21 [Trial 1], 2/21 [Trial 2], and 1/21 [Trial 3]) exceeded the acceptable level before treatment, while 8/63 (12.7%) samples (1/21 [Trial 1], 3/21 [Trial 2], and 4/21 [Trial 3]) exceeded the acceptable level after treatment (Fig. 2b, d, and f). The laboratory standard operating procedure requires benches to be disinfected after use. Our data suggest that the application of a biocidal SAQAS treatment does not offer any additional advantage for surfaces that are routinely disinfected, such as laboratory benchtops, or those that are infrequently contaminated.

### SAQAS-A is not effective for 30 days as a biocidal floor treatment

**Fig 3:**
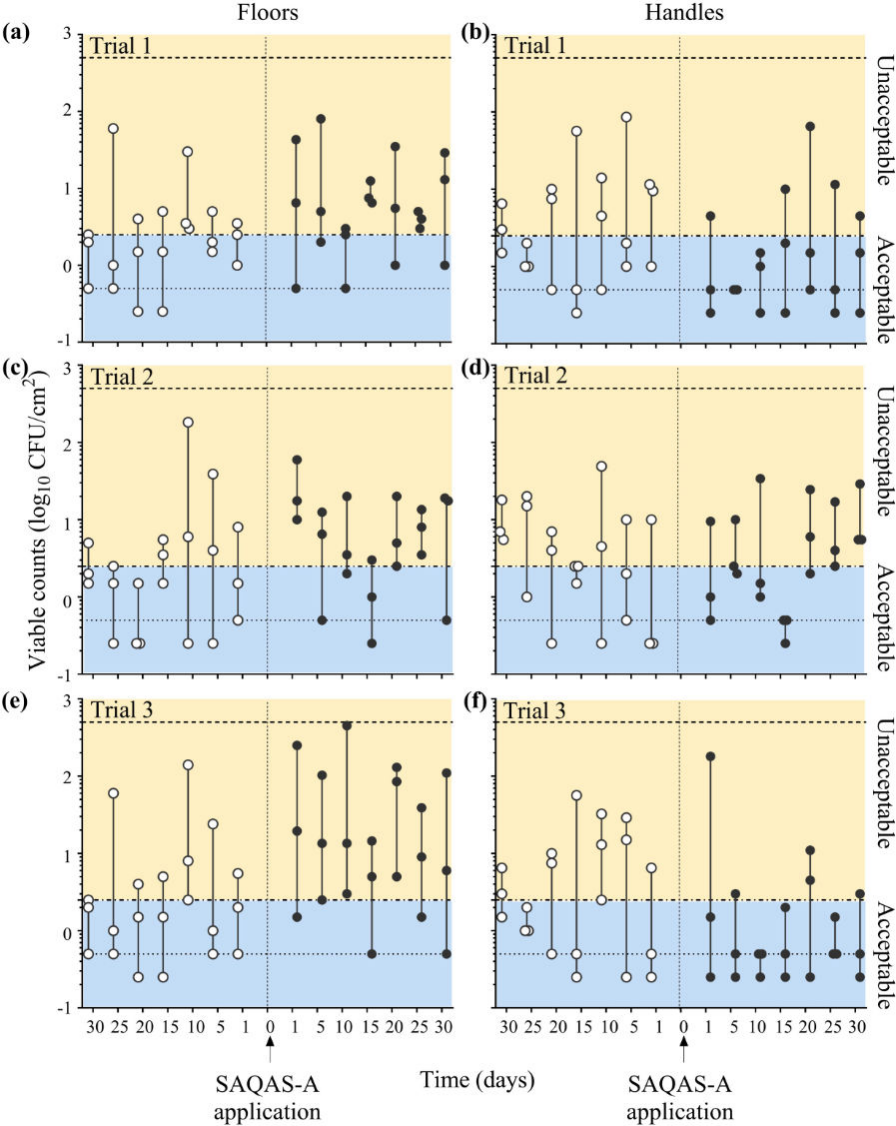
Bacterial counts recovered from floors and handles before and after the application of SAQAS-A. Data presented as individual viable counts (log_10_ CFU/cm^2^) recovered at regular intervals for 30 days before (open circles) and after (closed circles) the application of SAQAS-A to floors (a/c/e) and handles (b/d/f). Experiments were performed over three trials (Trial 1 [**a/b**], Trial 2 [**c/d**], and Trial 3 [**e/f**]). Dotted lines indicate the upper and lower limits of detection. Blue boxes (labeled “Acceptable”) denote viable counts below the acceptable level of 2.5 CFU/cm^2^; yellow boxes (labeled “Unacceptable”) denote viable counts above this level.

We determined the microbial burden present on floor surfaces before and after SAQAS-A application (Fig. 3a, c, and e). Our data show there was no reduction in recovered CFU due to the biocide treatment. In contrast, the number of samples in which the microbial burden exceeded the acceptable level of 2.5 CFU/cm^2^ was greater after biocide application (46/63 samples [73%]; 15/21 [Trial 1], 15/21 [Trial 2], and 16/21 [Trial 3]) than before (23/63 samples [37%]; 8/21 [Trial 1], 8/21 [Trial 2], and 7/21 [Trial 3]) (Fig. 3a, c, and e).

Cadnum et al. recently demonstrated that the amount of disinfectant applied to a surface can vary significantly depending on the method of application (31). They hypothesized that this may explain the discrepancy between *in vitro* results and real-world efficacy. In our study, we applied sufficient SAQAS-A to wet the sample areas thoroughly, allowed them to dry, and then repeated these steps a further two times. We have previously demonstrated that when applied in this way to glass and low-density polyethylene surfaces, SAQAS-A was active against model Gram-positive and Gram-negative species and antibiotic-resistant isolates (27). In this study, we observed that SAQAS-A-treated areas attracted more dust than untreated areas, which may explain the higher microbial burdens, as dust particles provide a surface for microbes to adhere to (32–34) and would form a barrier between the bacteria and the SAQAS layer. We believe this should be investigated further.

### SAQAS-A may offer some advantage as a biocidal treatment of high-touch, infrequently cleaned surfaces

We determined the microbial burden present on high-touch, infrequently cleaned incubator handles before and after SAQAS-A application (Fig. 3b, d, and f). Our data show that these high-touch surfaces frequently exceeded the highest acceptable level of 2.5 CFU/cm^2^ during the 30-day period prior to each trial (31/63 samples [49%]; 10/21 [Trial 1], 11/21 [Trial 2], and 10/21 [Trial 3]) (Fig. 3b, d, and f). However, during the testing of SAQAS-A, for 2/3 trials, the number of samples exceeding the highest acceptable level was halved (10/42 samples [24%]; 5/21 [Trial 1] and 5/21 [Trial 3]) (Fig. 3b and f). For Trial 2, the number of samples exceeding the acceptable level was similar before and after treatment (11/21 and 10/21, respectively) (Fig. 3d). This suggested that SAQAS-A may offer some advantages as a biocidal treatment for high-touch surfaces that are not routinely cleaned, such as portable equipment. However, a closer inspection of the data obtained from each individual handle (see Fig. S1 at https://doi.org/10.17608/k6.auckland.29983177) revealed only a marginal benefit, which varied by handle and trial. For example, in Trial 3, there was a reduction in CFU for the most contaminated handle after SAQAS-A application; however, bacterial levels remained above the allowable limit on three of the seven sampling times (see Fig. S1G).

### SAQAS-A had no antimicrobial effect within 5 or 30 days of application

The practicalities of testing SAQAS-A in a working laboratory meant that trials were performed at different times of the year and using different sampling sites. We analyzed the microbial burden data using two generalized linear mixed models (GLMM) to investigate whether there were any significant differences in microbial burden before and after the application of SAQAS-A for the different types of surfaces and between trials. We also tested for interactions between these variables. In the first model, we compared all data obtained from the pre-SAQAS-A application with all data obtained post-application (Table 1; also see Table S3 at https://doi.org/10.17608/k6.auckland.29983177). For the second model, we investigated whether SAQAS-A exhibited any antimicrobial activity within the first 5 days of application (Table 1; Table S3). Our analyses indicated that the SAQAS-A application had no antimicrobial effect. The first statistical model revealed that, overall, microbial counts were significantly higher after applying SAQAS-A than before (*P* = 0.005, GLMM with Šidák correction for multiple comparisons) (Table 1). Similarly, the second model showed that microbial burdens were significantly higher within the first 5 days after SAQAS-A application (*P* = 0.040, GLMM with Šidák correction for multiple comparisons) but then decreased back to pre-intervention levels (Table 1).

**TABLE 1:** Statistical analyses (pairwise contrasts) of microbial burden data^*f*^

Intervention pairwise contrasts	CE^*a*^	SE^*b*^	t	DF^*c*^	Sig.^*d*^	95% CI^*e*^	
						Lower	Upper
Model 1							
Post-SAQAS to pre-SAQAS	2.528	0.896	2.82	486	**0.005**	0.768	4.288
Model 2							
>5 days to ≤5 days post-SAQAS	−5.481	2.407	−2.28	480	**0.046**	−10.881	−0.080
>5 days post-SAQAS to pre-SAQAS	0.305	0.615	0.50	480	0.621	−0.905	1.514
≤5 days post-SAQAS to pre-SAQAS	5.785	2.336	2.48	480	**0.040**	0.187	11.384

^*a*^ CE, contrast estimate.

^*b*^ SE, standard error.

^*c*^ DF, degrees of freedom.

^*d*^ Sidak (adjusted).

^*e*^ CI, confidence intervals (approximate).

^*f*^ Significant values shown in bold.

### SAQAS-A is easily removed by wiping with water or 70% ethanol

**Fig 4:**
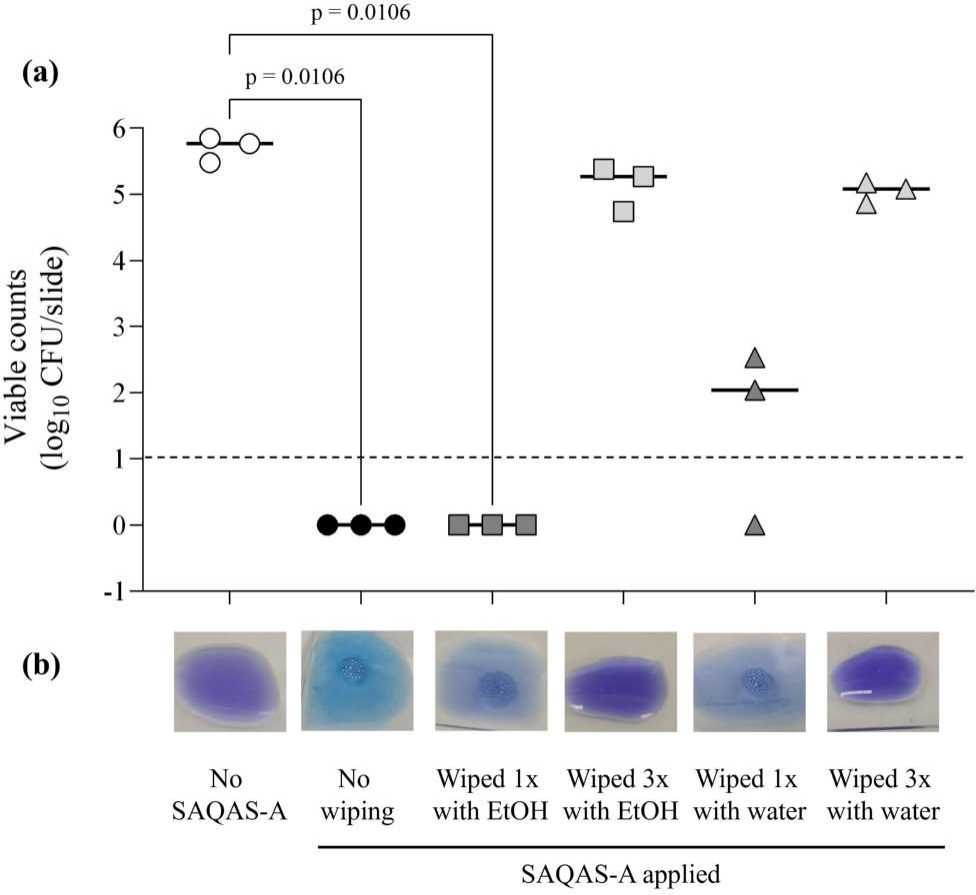
SAQAS-A was removed from glass by wiping with ethanol or water. Glass microscope slides were treated with SAQAS-A and durability investigated after cleaning with ethanol or glass by testing for residual antibacterial activity (**a**) and using a colorimetric assay in which bromophenol blue changes from dark blue/purple to light blue in the presence of a quaternary ammonium such as SAQAS-A (**b**). Data is presented as dot plots of viable counts (log_10_ CFU/slide) of *Staphylococcus aureus* recovered 30 min after inoculation of untreated glass microscope slides (open circles) or slides treated with SAQAS-A (filled shapes). SAQAS-A-treated slides were either left undisturbed (black circles), wiped once with 70% ethanol (dark gray squares), wiped three times with 70% ethanol (light gray squares), wiped once with water (dark gray triangles), or wiped three times with water (light gray triangles) (*n* = 3 per group). Solid lines indicate median values. The dotted line indicates the limit of detection. Data were analyzed using a Kruskal-Wallis test with Dunn’s test for multiple comparisons.

Calfee et al. have previously reported that the efficacy of numerous antimicrobial surface coatings was reduced to near zero after being cleaned with hypochlorite or a quaternary ammonium compound disinfectant (35). To investigate whether routine disinfection of the treated surfaces was a factor in the poor long-term protection we observed, we tested whether SAQAS-A could be removed from glass microscope slides by wiping with either water or 70% ethanol. In the presence of a quaternary ammonium, bromophenol blue changes from dark blue/purple to light blue, so we used this colorimetric assay to rapidly assess the presence or absence of SAQAS-A on cleaned slides, as well as testing them for activity against *Staphylococcus aureus*. Our data show that wiping SAQAS-A-treated glass slides three times with ethanol or water was sufficient to remove the product and any anti-*S*. *aureus* activity (Fig. 4). Indeed, just one wipe with water was enough to reduce the anti-*S*. *aureus* efficacy of the SAQAS-A coating. This suggests that regular cleaning with liquid disinfectants, or even water, erodes SAQAS-A from surfaces.

### Conclusion

Antimicrobial surfaces are used to reduce the microbial burden on surfaces and prevent the transmission of pathogens. It is crucial to verify the success of this approach using real-world environments (24, 36). We have tested a commercially available contact-dependent SAQAS-type biocide formulation (SAQAS-A) and demonstrated that it was unable to protect surfaces for either 5 or 30 days in a real-world setting that included routine chemical disinfection of surfaces. SAQAS-A may have the potential to protect surfaces that are not routinely cleaned, such as portable equipment.

Our previously published work demonstrated that SAQAS-A treatment protected surfaces in assays using treated glass and low-density polyethylene carriers (27). The lack of activity of the SAQAS-A treatment within a working microbiology laboratory was disappointing. Gram-positive bacteria, including *Staphylococcus* spp., were able to survive on surfaces treated with SAQAS-A, indicating that routine cleaning of surfaces reduces the product’s potency. Future work should investigate the long-term chemical stability and durability of biocides on surfaces under conditions that better mimic the conditions of their intended use.

## MATERIALS AND METHODS

A commercially available, spray-on biocide formulation (Zoono Z-71 Microbe Shield Surface Sanitizer) (Elitepac NZ Ltd.) was used and is referred to as SAQAS-A. This was applied by spraying surfaces until wet, then allowing them to dry before repeating the process a further two times, delivering approximately 500 µL SAQAS-A per 5 cm^2^. Surface disinfectants were Trigene Advance (Invitro Technologies, New Zealand), used at a 1:100 dilution in water, and ethanol, used at 70% by volume in water.

### Environmental sampling

Three high-traffic floor areas, three routinely used bench areas, three frequently touched handles, and three glass surfaces were designated as sampling sites within the PC2 laboratory (Fig. 3). Each designated sampling area was 20 cm^2^. To maintain consistency, we used a stencil and marked the sampling area using a marker pen to ensure repeatability and standardization across samples. The chosen areas (see Table S1 at https://doi.org/10.17608/k6.auckland.29983177) were routinely cleaned according to local laboratory procedures during the trial period. Floors were cleaned twice a week using Trigene Advance, and bench surfaces were cleaned after every period of microbiological work using Trigene Advance, followed by 70% ethanol. Door handles were cleaned once a week using 70% ethanol. Incubator handles were cleaned less regularly. There was no routine cleaning of window-glass surfaces. Trigene Advance was applied for a contact time of 10 min and 70% ethanol until it had evaporated (approx 1 min).

Cotton swabs were purchased from Global Science & Technology, Auckland, New Zealand, and sterilized in-house by autoclaving. Microorganisms were released from swabs in either Peptone Water (Fort Richard, Auckland, New Zealand) or Letheen Broth (Merck Millipore Corporation, USA). Each designated area was sampled between 11:30 a.m. and 1:30 p.m. every 5 days over 30-day periods before and after SAQAS-A treatment, and bacterial enumeration on agar plates was used to determine the microbial burden. Experiments were repeated on three separate occasions (Trials 1, 2, and 3). For sampling, designated areas were swabbed with a sterile cotton swab that had first been moistened in sterile saline (0.85% wt/vol NaCl) and then pressed against the inside wall of the tube to remove any excess liquid. Surfaces were swabbed horizontally, vertically, and then diagonally for approximately 10 passes in each direction, with the swab being rotated axially between the thumb and forefinger to ensure all sides of the swab were used. Sufficient pressure was applied to the surface through the swab to cause the swab shaft to begin to bend. The swab heads were then placed into a bottle containing 10 mL of peptone water (Trial 1) or 2 mL Letheen broth (Trials 2 and 3), and the shaft was snapped off aseptically against the inner wall of the bottle, leaving the cotton head in the bottle. Letheen broth was used as a precaution to neutralize any biocide also removed from the surface that we speculated would reduce the microbial burden recovered on the pour plates. The capped bottles were vortexed for 3 × 20 s with 20 s of rest between each vortex. Aliquots of 100 µL were plated onto Difco Tryptic Soy Agar (TSA), incubated at 28°C for 2 days, and CFU counted.

### Bromophenol blue assay

Glass microscope slides were cleaned with 70% ethanol, and SAQAS-A was applied as described above. Untreated slides were used as a control. To ensure treated slides were cleaned consistently, a disposable paper towel (Tork Xpress, Essity Hygiene and Health) was moistened with sterile MilliQ water or 70% ethanol and wrapped around the base of a 500 mL bottle filled with water until it weighed 500 g. For the one-wipe protocol, microscope slides were held on a sterile surface, and the bottle was pushed once across the surface. For the three-wipe protocol, the slide was allowed to dry between wipes, and a fresh, moistened paper towel was used for each wipe.

To test whether wiping the slides removed SAQAS-A, we repeatedly pipetted 100 µL of an 83 ppm bromophenol blue solution in 0.1 M sodium phosphate buffer pH 7.0 (37) onto each slide and left for 15–30 s before photographing. In the presence of a quaternary ammonium, bromophenol blue changes from dark blue/purple to light blue. We also tested the antibacterial efficacy of SAQAS-A-treated slides after wiping. Slides were inoculated with 10 µL of approximately 10^6^ CFU of *Staphylococcus aureus* ATCC 6538, prepared by diluting a culture grown overnight in Difco Tryptic Soy Broth in phosphate-buffered saline. Drops were allowed to dry and left for 30 min. Viable cells were recovered by repeated pipetting of the dried inoculum with casein peptone lecithin polysorbate broth (CPLPB) (Merck) and enumerated by plating onto TSA after dilution in CPLPB and incubating at 37°C overnight.

### Generalized linear mixed models

The data obtained from testing the real-world efficacy of SAQAS-A on laboratory surfaces were analyzed using two GLMMs using IBM SPSS Statistics for Windows (Version 26.0) (IBM Corp., Armonk, NY). A mixed model was used because multiple measurements were taken from the same areas, and this type of model considers the measurements within each area as being related (38). The models incorporated a negative binomial distribution with logarithmic links and repeated measures. A Sidak sequential adjustment was applied to all post hoc pairwise comparisons to adjust for multiple testing (39). Factors included in model 1 were intervention (pre/post-SAQAS-A treatment), surface type (bench/floor/glass/handles), and trial (1–3). The same factors were included in model 2, but this model also tested the strength of the intervention in the first 5 days (post ≤5 days) versus longer-term (post >5 days: 10-to-30 days).

## Data Availability

The data underlying this article are available in Table S1 at https://doi.org/10.17608/k6.auckland.29983177 and on Figshare (https://figshare.com/s/fd3c3511fcc2aadb3e30).
